# Protective Effect of γ-Aminobutyric Acid Against Chilling Stress During Reproductive Stage in Tomato Plants Through Modulation of Sugar Metabolism, Chloroplast Integrity, and Antioxidative Defense Systems

**DOI:** 10.3389/fpls.2021.663750

**Published:** 2021-10-18

**Authors:** Ola H. Abd Elbar, Amr Elkelish, Gniewko Niedbała, Reham Farag, Tomasz Wojciechowski, Soumya Mukherjee, Ayman F. Abou-Hadid, Hussien M. El-Hennawy, Ahmed Abou El-Yazied, Hany G. Abd El-Gawad, Ehab Azab, Adil A. Gobouri, Nihal El Nahhas, Ahmed M. El-Sawy, Ahmed Bondok, Mohamed F. M. Ibrahim

**Affiliations:** ^1^Department of Agricultural Botany, Faculty of Agriculture, Ain Shams University, Cairo, Egypt; ^2^Department of Botany, Faculty of Science, Suez Canal University, Ismailia, Egypt; ^3^Department of Biosystems Engineering, Faculty of Environmental and Mechanical Engineering, Poznań University of Life Sciences, Poznań, Poland; ^4^Department of Botany, Jangipur College, University of Kalyani, West Bengal, India; ^5^Department of Horticulture, Faculty of Agriculture, Ain Shams University, Cairo, Egypt; ^6^Department of Food Science and Nutrition, College of Science, Taif University, Taif, Saudi Arabia; ^7^Department of Chemistry, College of Science, Taif University, Taif, Saudi Arabia; ^8^Department of Botany and Microbiology, Faculty of Science, Alexandria University, Alexandria, Egypt; ^9^Department of Climate Modification, Central Laboratory for Agriculture Climate, Agriculture Research Center, Giza, Egypt; ^10^Department of Plant Pathology, Faculty of Agriculture, Ain Shams University, Cairo, Egypt

**Keywords:** tomato (*Solanum lycopersicum* L.), gamma-aminobutyric acid, chilling stress, chloroplast ultrastructure, oxidative stress, antioxidants, fruit yield

## Abstract

Despite the role of γ-aminobutyric acid (GABA) in plant tolerance to chilling stress having been widely discussed in the seedling stage, very little information is clear regarding its implication in chilling tolerance during the reproductive stage of the plant. Here, we investigated the influence of GABA (1 and 2mM) as a foliar application on tomato plants (*Solanum lycopersicum* L. cv. Super Marmande) subjected to chilling stress (5°C for 6h/day) for 5 successive days during the flowering stage. The results indicated that applied GABA differentially influenced leaf pigment composition by decreasing the chlorophyll a/b ratio and increasing the anthocyanin relative to total chlorophyll. However, carotenoids were not affected in both GABA-treated and non-treated stressed plants. Root tissues significantly exhibited an increase in thermo-tolerance in GABA-treated plants. Furthermore, applied GABA substantially alleviated the chilling-induced oxidative damage by protecting cell membrane integrity and reducing malondialdehyde (MDA) and H_2_O_2_. This positive effect of GABA was associated with enhancing the activity of phenylalanine ammonia-lyase (PAL), catalase (CAT), superoxide dismutase (SOD), and ascorbate peroxidase (APX). Conversely, a downregulation of peroxidase (POX) and polyphenol oxidase (PPO) was observed under chilling stress which indicates its relevance in phenol metabolism. Interesting correlations were obtained between GABA-induced upregulation of sugar metabolism coinciding with altering secondary metabolism, activities of antioxidant enzymes, and maintaining the integrity of plastids’ ultrastructure Eventually, applied GABA especially at 2mM improved the fruit yield and could be recommended to mitigate the damage of chilling stress in tomato plants.

## Introduction

Tomato (*Solanum lycopersicum* L.) is a cosmopolitan economical vegetable crop cultivated worldwide, distributed in diverse climate zones. This leads to cultivation exposing the plant to various environmental stresses (Chaudhary et al., 2019). Most cultivated genotypes are sensitive to low temperatures in all growth stages (Foolad and Lin, 2000). As tomato is a warm-season crop which grows well in the range of 25–28°C, growth will be limited when the plants are exposed to low temperature (5–13°C; Aghdam et al., 2012).

In recent decades, global climate change has caused sudden, variable, and extreme weather changes repeatedly leading to high losses of crop productivity and yield (Alam et al., 2019; Ahanger et al., 2020; Ahmad et al., 2020; Jan et al., 2020; Kaya et al., 2020). Particularly, chilling stress is deleterious to the growth and development of crops that have originated in temperate zones (Zhang et al., 2004; Ma et al., 2018). Chilling stress causes a rapid and synchronized change in the thermodynamic microclimate of every plant cell, including in the molecular makeup of all organelles. Enzymatic reactions are slowed due to a reduction in the diffusion rates of substrates (Kratsch and Wise, 2000). This in turn will affect photosynthesis performance including carbon fixation, stomatal conductance, electron transport, and the functional roles of PSI and PSII (Allen and Ort, 2001; Sun et al., 2008; Liu et al., 2012; Zhang et al., 2014). Consequently, this has resulted in the generation of reactive oxygen species (ROS; Ahmad et al., 2010, 2019; Kohli et al., 2019) and instantly caused damage for membranes, resulting in an interruption in transport processes across membranes and membrane-bound enzyme activity (Ahmad et al., 2010, 2019; Zhang et al., 2014, 2015; Kohli et al., 2019). Moreover, chilling causes ultrastructural injures particularly for the chloroplasts, which we are considering the most severely impacted organelles. The symptoms observed are swelling and disorganization of the chloroplast and dilation of thylakoids thus leading to a subsequent increase in plastoglobule numbers (Musser et al., 1984; Zbierzak et al., 2013; Karim et al., 2014). Various symptoms of chilling stress include grana disorganization, changes in thylakoid and chloroplast membrane followed by accumulation of lipid droplets, and darkening of the stroma (Musser et al., 1984; Kratsch and Wise, 2000; Zbierzak et al., 2013; Karim et al., 2014). However, plants resistant to chilling stress exhibit a reduction in the size and number of starch grains (Kratsch and Wise, 2000; Zhuang et al., 2019), and more condensed grana disks are present (Garstka et al., 2007). Structural and physiological changes are closely related to the accumulation of ROS in the chloroplast, which is considered the main site for generating ROS under unfavorable stress conditions thereby damaging the photosynthetic apparatus (Partelli et al., 2011; Kim, 2020). These injuries are proportional to the length of time spent at the damaging temperature and the physiological age of the plant.

Various priming molecules have been suggested to induce chilling stress tolerance in plants (Malekzadeh et al., 2014; Han et al., 2016; Zhao et al., 2016). γ-aminobutyric acid (GABA), is an important non-proteinogenic amino acid present in very low levels in plant tissue and involved in some physio-biochemical functions related to plant growth and development (Sita and Kumar, 2020). It can be rapidly accumulated in plant tissue as a response to several biotic and abiotic stresses (Roberts, 2007). Exogenous application of GABA plays a substantial role in the alleviation of a wide array of abiotic stresses such as drought (Abd El-Gawad et al., 2020), salinity (Wu et al., 2020), chilling (Malekzadeh et al., 2014; Wang et al., 2014), heavy metals (Seifikalhor et al., 2020), low light, and nitrogen starvation (Kinnersley and Lin, 2000). These effects can be explained *via* regulating osmotic pressure, pH scale, H^+^ in cytosol, C and N metabolism, and scavenging free radicals (Gilliham and Tyerman, 2016). Consequently, exogenous applied-GABA can enable plants to enhance their photosynthetic capacity by affecting the level of ROS by altering the activities of antioxidant enzymes and maintaining the membrane integrity (Aghdam et al., 2015).

Although several studies have confirmed that GABA can alleviate chilling injuries in tomato plants, all have focused on the seedling stage (Aghdam et al., 2012; Malekzadeh et al., 2014). To date, insufficient information exists on the role of GABA in alleviating chilling injuries during the reproductive and fruit set stages. In this study, we provided evidence of the implication of GABA in mitigation of the chilling-induced damages in tomato plants through the protection of the chloroplast ultrastructure, altering several primary and secondary metabolism pathways and reducing the oxidative damage during reproductive and fruit set stage. These findings could be further elucidating the mechanism of GABA in plant tolerance to chilling stress.

## Materials and Methods

### Experimental Design and Growth Conditions

**Figure 1:**
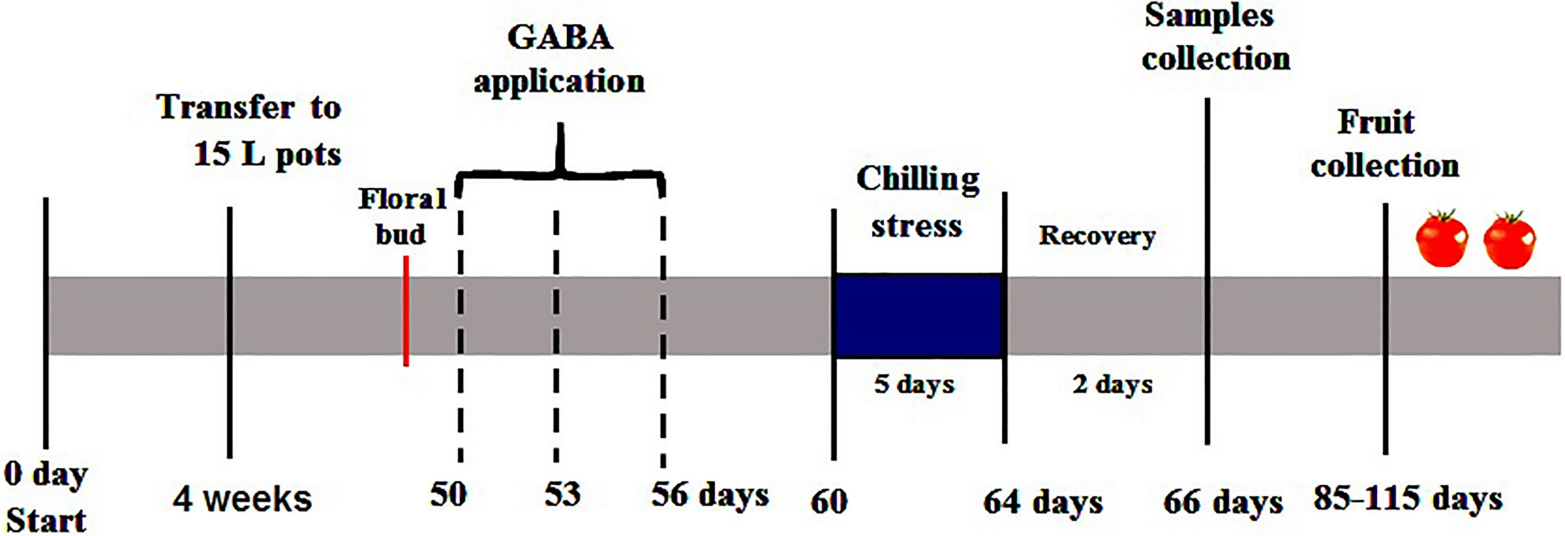
The timeline infographic for the treatments of GABA as a foliar application and the samples’ collection of tomato plants subjected chilling stress in the reproductive stage.

Tomato seeds (*Solanum lycopersicum* L. cv. Super Marmande) were sterilized for 4min with 0.7% (w/v) NaOCl and washed with distilled water several times. Seeds were germinated in trays with 50 individual cells (4×4×6cm) containing peat and vermiculite (3/1v/v) at temperature (25/18°C) and a 16/8h light/dark cycle. About 4-week-old tomato seedlings at the four-leaf stage homogenized in shape and size were grown into 15-L plastic pots filled with sterilized sandy-loamy soil (2:1 w/w) during the period from 10th November 2019 to 4th March 2020 in a greenhouse at the Department of Plant Pathology, Faculty of Agriculture, Ain Shams University, Cairo, Egypt. Average air temperature and relative humidity (Table 1) of the greenhouse were recorded by digital Thermo/hygrometer Art placed in the middle of the greenhouse (No.30.5000/30.5002, TFA, Germany). All pots were irrigated with half-strength Hoagland’s solution 2–3 times a week according to the growth stage of the plants. When the first floral bud started emerging, the plants were divided into three groups; each plant in the first group was sprayed three times with 15ml distilled water plus 0.05% Tween-20, (V/V) as a non-ionic surfactant (one time every 3days). Whereas, each plant in the second and third group was sprayed every time with 15ml of 1 and 2mM γ-aminobutyric acid, GABA (Sigma-Aldrich, Munich, Germany) plus 0.05% Tween-20 (V/V) respectively. The first group was divided into the Control subgroup (normal conditions), and the chilling subgroup (exposed to 5°C for 6h/day for 5 successive days). The second and third groups were divided into GABA subgroup under normal conditions, and GABA subgroup with chilling stress. Subsequently, all subgroups of chilling stressed plants were transferred to the normal conditions for 2days of recovery. Then, the leaves were collected and stored at −80°C for further physiological and biochemical examination. The experimental layout was a complete randomized Design (CRD) with three replicates. Each replicate contained 30 individual plants (five plants×six treatments). Six plants from each treatment (two plants/replicate) were left to the end of the season to evaluate the eventual fruit yield. The timeline infographic for the treatments and samples’ collection was shown in Figure 1.

**Table 1:** Summary of the monthly mean climate condition, maximum (T_max_) and minimum (T_min_), mean (T_ave_) daily temperatures and relative humidity (RH), inside the greenhouse.

Month	T_max_	T_min_	T_ave_	RH (%)
November	32.15	19.32	25.74	72.25
December	27.55	15.30	21.43	76.31
January	24.95	14.50	19.74	79.84
February	29.73	15.63	22.68	74.14
March	30.11	18.54	24.33	75.77

### Determination of Leaf Pigments

Chlorophyll a, b, and total chlorophyll were determined as described by Costache et al. (2012) with some modification, small pieces of fresh leaves (0.5g) were submerged into 10ml pure acetone for 24h/4°C. The absorbance was measured at 645 and 663nm, respectively. The concentration was calculated using the following equations:


$$\begin{array}{l} \mathrm{Chlorophyll}\;a\;\left(\mathrm{mg}/g\;\mathrm{FW}\right)\\ =11.75\;A662-2.350\;A645\times \left(V/1000\times W\right). \end{array}$$

$$\begin{array}{l} \mathrm{Chlorophyll}\;b\;\left(\mathrm{mg}/g\;\mathrm{FW}\right)\\ =18.61\;A645-3.960\;A662\times \left(V/1000\times W\right). \end{array}$$

Where A is the absorbance at 645 and 663nm, V is the Final volume of chlorophyll extract in pure acetone and W is the fresh weight of tissue extract. Total chlorophyll was calculated as the sum of chlorophyll a+b.

Carotenoids were quantified using the acetone and petroleum ether method as described by De Carvalho et al. (2012) using the following formula:


$$\begin{array}{l} \mathrm{Carotenoids}\;\left(\mathrm{mg}/g\;\mathrm{FW}\right)\\ =A_{450}\;x\;V\;\left(\mathrm{ml}\right)\;x\;10/\left[A^{1\%}{}_{\mathrm{1cm}}\;x\;W\;\left(g\right)\right]. \end{array}$$

Where A_450_=Absorbance at 450nm, V=Total extract volume; W=sample weight; A^1%^_1cm_=2,592 (β-carotene coefficient in petroleum ether). Anthocyanin was determined according to Harvaux and Kloppstech (2001). A fresh weight (0.4g) was ground into 10ml acidified methanol (99 MeOH: 1 HCl; v/v). The samples were centrifuged (4,000rpm/10min), then the absorption was recorded on a spectrophotometer at 530nm, the concentration of anthocyanin was expressed as ∆ O.D/100mg FW.

The specific wavelengths for all estimated leaf pigments were determined using UV–visible spectrophotometer (UV-1601PC; Shimadzu, Tokyo, Japan).

### Determination of Reducing/Non-reducing and Total Soluble Sugars

Total soluble sugars were estimated using the colorimetric method of anthrone and sulfuric acid (Yemm and Willis, 1954), whereas, the reducing sugars were determined using the method of 3, 5-Dinitrosalicylic acid (DNS) as described by Miller (1959). Non-reducing sugar content was estimated using the difference between the total soluble sugar content and the reducing sugars.

### Determination of Leaf Oxidative Damage

Hydrogen peroxide was quantified by the colorimetric method of potassium iodide as described by Velikova et al. (2000). A fresh weight (0.5g) of leaf tissues was homogenized in 3ml of 1% (w/v) tri-chloroacetic acid (TCA). The homogenate was centrifuged at 10,000rpm at 4°C for 15min. Subsequently, 0.75ml of the supernatant was added to 0.75ml of 10mM K-phosphate buffer (pH 7.0) and 1.5ml of 1M KI. The mixture was measured at 390nm using a spectrophotometer (UV-1601PC; Shimadzu, Tokyo, Japan) and the concentration was calculated according to a previously prepared standard curve.

Lipid peroxidation as malondialdehyde (MDA) was determined using thiobarbituric acid (TBA) as described by Heath and Packer (1968) with some minor modifications. A fresh weight of leaves (0.2g) was homogenized with 0.1% trichloroacetic acid homogenates (TCA; m/v) and 5% PVP (m/v). The homogenate was centrifuged at 5000rpm for 15min. Around 3ml of the supernatant were added to the reaction medium consisting of 0.5% (m/v) thiobarbituric acid (TBA) and 10% (w/v) TCA. The mixture was heated in boiling water for 30min then cooled rapidly in ice. The absorbance of reddish color was measured at 535 and 600nm using a spectrophotometer (UV-1601PC; Shimadzu, Tokyo, Japan). The concentration of the MDA/TBA complex was calculated using the following equation:


$$\mathrm{MDA}\;\left(\mathrm{nmol}\cdot g^{-1}\;\mathrm{FW}\right)=\left(A535-A600\right)/\varepsilon .$$

Where ε is the extinction coefficient=155mM^−1^ cm^−1^.

Electrolyte leakage was measured as a percentage between readings of EC meter (DOH-SD1, TC-OMEGA, United States/Canada) before and after killing leaf tissues by autoclave at 120°C for 20min as described by Singh et al. (2008) with some minor modifications. Eight leaf discs (2cm diameter) were taken by a cork borer, cleaned well and incubated in 10ml deionized water for 24h on a shaker. The EC of the solution was measured twice, the first one immediately after the incubation period and the second after killing the leaf tissue by autoclave.

### Determination of Root Thermotolerance

After sampling, roots were collected and washed with tap water several times. The root tissues were cut into small pieces and incubated in darkness for 24h/37°C with a solution containing 0.6% 2,3,5 triphenyltetrazolium chloride (TTC). The red color of formazan was extracted in 95% ethanol and the absorbance was observed on a spectrophotometer (UV-1601PC; Shimadzu, Tokyo, Japan) at 490nm (Xu and Huang, 2008).

### Total Soluble Protein and Enzyme Assays

To prepare the extraction of enzyme and soluble proteins, fresh leaves (0.5g) were homogenized in 4ml 0.1M sodium phosphate buffer (pH 7.0) containing 1% (w:v) polyvinylpyrrolidone (PVP) and 0.1mM EDTA, centrifuged at 10,000×*g* for 20min at 4°C and then the supernatant was used for assays. Soluble proteins were evaluated by the method of Bradford (1976). All studied enzyme activities and protein concentration in the crude enzyme extract were measured using a spectrophotometer (UV-1601PC; Shimadzu, Tokyo, Japan) as follows:

Superoxide dismutase (SOD) assay was based on the method described by Beyer and Fridovich (1987). The reaction mixture with a total volume of 3ml contained 100μl crude enzyme, 50mM phosphate buffer (pH 7.8), 75μM NBT, 13mM L-methionine, 0.1mM EDTA, and 0.5mM riboflavin. The reaction was initiated by the addition of riboflavin then the reaction mixture was illuminated for 20min with a 20W fluorescent lamp. One unit of enzyme activity was defined as the amount of enzyme required to result in a 50% inhibition in the rate of nitro blue tetrazolium (NBT) reduction at 560nm.

Catalase (CAT) activity was measured by monitoring the decrease in absorbance at 240nm as described by Cakmak et al. (1993). The reaction mixture with a total volume of 3ml contained 15mM H_2_O_2_ in 50mM phosphate buffer (pH=7). The reaction was initiated by adding 50μl crude enzyme. The activity was calculated from the extinction coefficient (ε=40mM^−1^ cm^−1^) for H_2_O_2_. One unit of enzyme activity was defined as the decomposition of 1μmol of H_2_O_2_ per minute.

The activity of ascorbate peroxidase (APX) was determined according to Nakano and Asada (1981). The decrease of absorbance at 290nm was monitored for 3min. The reaction mixture with a total volume of 3ml included 100μl crude enzyme, 50mM phosphate buffer (pH 7), 0.1mM EDTA, 0.5mM ascorbic acid, and 0.1mM H_2_O_2_. The reaction was initiated by the addition of H_2_O_2_. One unit of enzyme activity was defined as the amount of enzyme required for oxidation of 1μmol of ascorbate per minute. The rate of ascorbate oxidation was calculated using the extinction coefficient (ε=2.8mm^−1^ cm^−1^).

Polyphenol oxidase (PPO) activity was determined according to Oktay et al. (1995). The reaction mixture consisted of 100μl crude enzyme, 600μl catechol, and 2.3ml phosphate buffer (0.1M, pH 6.5). The absorbance at 420nm was recorded at zero time and after 1min using a spectrophotometer.

Peroxidase (POX) activity was quantified by the method of Dias and Costa (1983) with some minor modifications. The assay mixture (100ml) contained 10ml of 1% (v/v) guaiacol, 10ml of 0.3% H_2_O_2_ and 80ml of 50mM phosphate buffer (pH=6.6). The volume of 100μl of the crude enzyme was added to 2.9ml of the assay mixture to start the reaction. The absorbance was recorded every 30s for 3min at 470nm.

The activity of phenylalanine ammonia-lyase (PAL) was determined as Trans-cinnamic acid as described by Lister et al. (1996). The PAL assay reaction consisted of 100μl crude extract and 900μl of 6μmol phenylalanine in 500mM tris-HCl buffer (pH 8.5). The mixture was incubated at 37°C for 1h and measured spectrophotometrically at 290nm. Trans-cinnamic acid was used as standard.

### Sample Preparation and Observation by Transmission Electron Microscopy

Small leaf pieces (~3mm×1.5mm) were cut and fixed in 3% glutaraldehyde rinsed in 0.1m phosphate buffer (pH=7.6) for 6h. at 4°C and post-fixed in 1% potassium permanganate solution for 5min at room temperature. The samples were dehydrated in an ethanol series ranging from 10 to 90% for 15min in each alcohol dilution and finally with absolute ethanol for 30min. Samples were infiltrated with EPON 812 epoxy resin (Sigma-Aldrich) and acetone through a graded series till finally in pure resin. For light microscopy, semi-thin leaf-cross sections were stained with toluidine blue and observed using LEICA light microscope model DM-500. Ultrathin sections were collected on copper grids. Sections were then double-stained in uranyl acetate followed by lead citrate. Stained sections were observed with a JEOL – JEM 1010 transmission electron microscope at 70kV at The Regional Center for Mycology and Biotechnology (RCMB), Al-Azhar University (Frankl et al., 2015).

### Statistics

One way ANOVA procedure was followed using SAS (1988) software. Means±SE were calculated from three replicates and the Duncan multiple range test (*p*≤0.05) was used to determine significant differences between means. Linear regression between some variables was also performed.

## Results

### Changes in Leaf Pigment Composition

**Figure 2:**
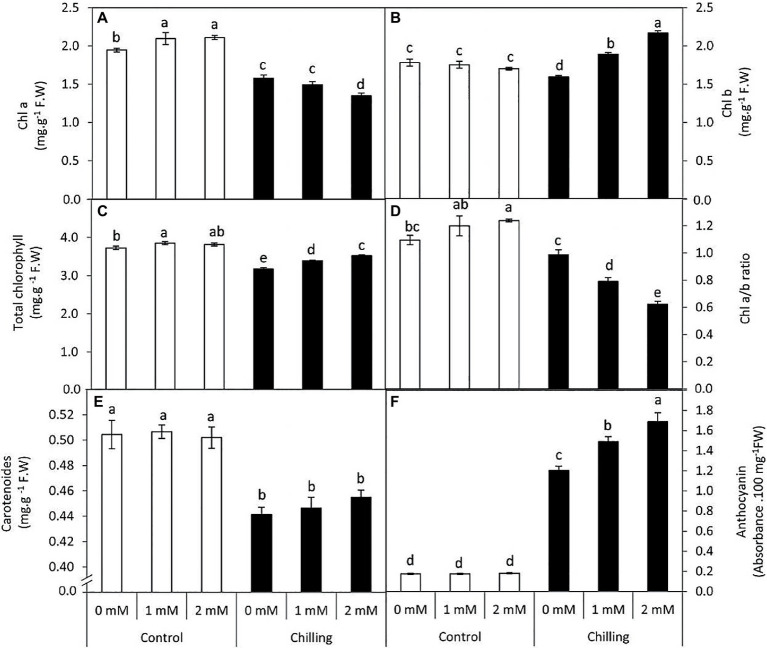
Effect of γ-aminobutyric acid (GABA) as a foliar application at 1 and 2mM on leaf pigments concentration of tomato plants cv “Super marmende” growing under normal (25°C/20°C day/night ) and chilling conditions (5 days/4ºC for 6 h/day) followed by 2 days recovery. **(A)**, chlorophyll a; **(B)**, chlorophyll b; **(C)**, total chlorophyll; **(D)**, chlorophyll a/b ratio; **(E)**, carotenoids, and **(F)**, anthocyanin. Data followed by the same letters ± SE are not significant according to Duncan multiple rang test at *P*≤0.05. The white columns refer to the control conditions and the black columns refer to the chilling stress.

Chilling-stress in tomato plants resulted in a significant (*p*≤0.05) decrease in chl a (19%) content in comparison with control plants while chl b exhibited marginal decrease (10.3%) during chilling stress (Figures 2A,B). Interestingly, exogenous GABA (1 and 2mM) application exerted contrasting effects on chla and chl b content during chilling stress. During Chilling stress chl a content underwent an obvious decrease in the presence of GABA treatments (23.3 and 30.7%) respectively. This decrease was accompanied by a steady increase in chl b (6.2 and 21.9%) content either in the presence of 1 or 2mM GABA, respectively. However, GABA application to non-stressed plants exhibited a marginal increase in chl a content, while changes were insignificant (*p*≤0.05) in chl b content. Thus, GABA applications (1 and 2mM) during chilling stress resulted in a distinct and significant (*p*≤0.05) decrease in chl a/b ratio (27.8 and 43.2% lower than the control) followed by a marginal increase in total chl content (Figures 2C,D). Contrastingly, chilling stress induced a significant (*p*≤0.05) decrease in the carotenoid content, which was observed to exhibit negligible effects upon GABA treatment (Figure 2E). Anthocyanin content exhibited a significant (*p*≤0.05) increase in the presence of chilling stress which all the more increases were observed in the presence of GABA (2mM; Figure 2F). Generally, under chilling stress, anthocyanin was the most affected pigment by the treatment of 2mM GABA (9.6-fold over the unstressed control plants) while this increase amounted to just 6.8 folds in the GABA untreated and chilling stressed plants.

### Changes in Leaf Sugar Metabolism

**Figure 3:**
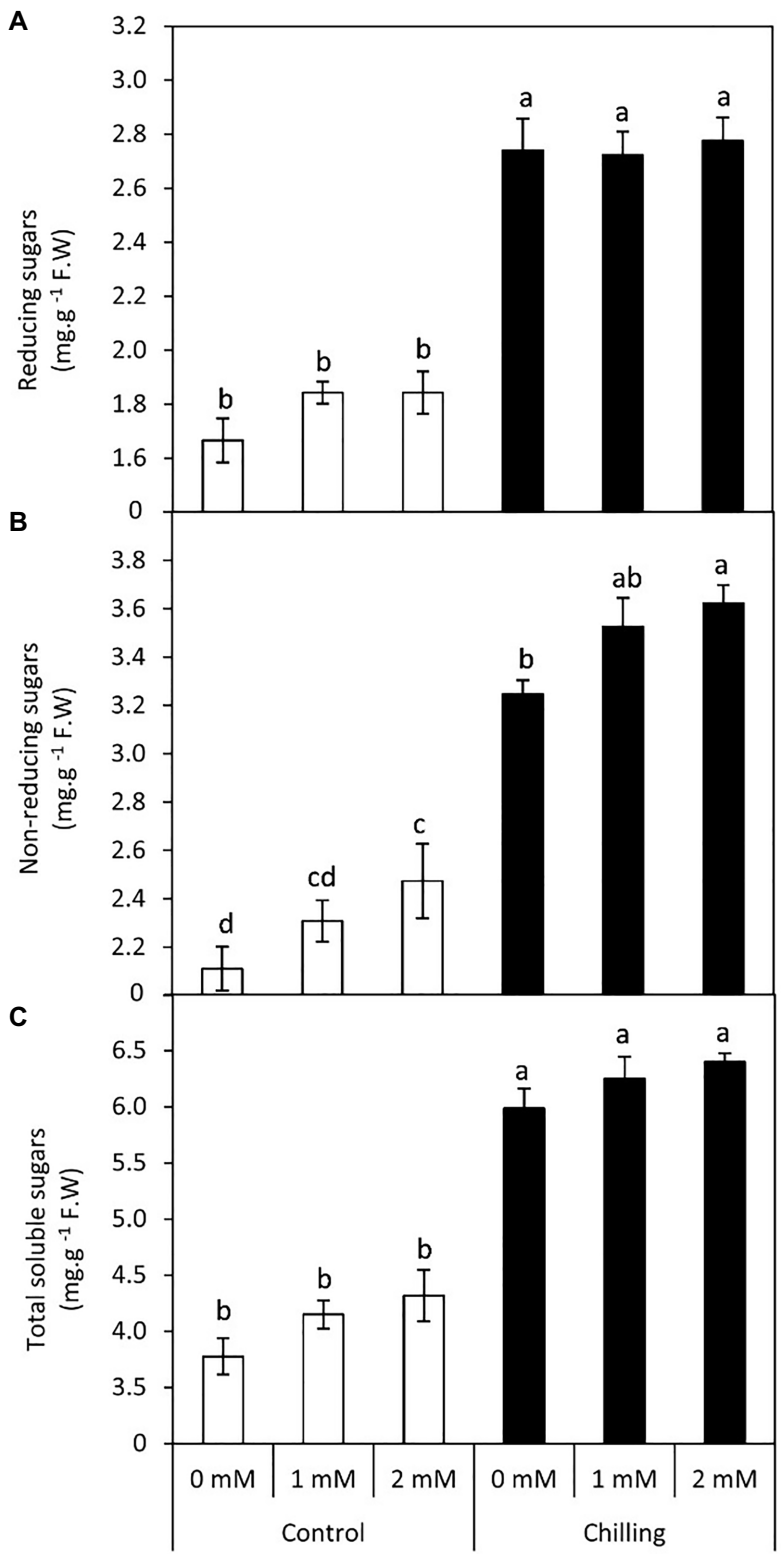
Effect of γ-aminobutyric acid (GABA) as a foliar application at 1 and 2mM on carbohydrate concentration of tomato plants cv “Super marmende” growing under normal (25°C/20°C day/night ) and chilling conditions (5 days/4ºC for 6h/day) followed by 2 days recovery. **(A)**, reducing sugars; **(B)**, non-reducing sugars and **(C)**, total soluble sugars. Data followed by the same letters ± SE are not significant according to Duncan multiple rang test at *P*≤0.05. The white columns refer to the control conditions and the black columns refer to the chilling stress.

In the present work, chilling stress resulted in a significant (*p*≤0.05) increase in reducing, non-reducing, and total soluble sugars (Figures 3A–C). Although exogenous GABA application marginally increased the levels of all three types of sugars, 2mM GABA exerted significant (*p*≤0.05) positive effects on the non-reducing sugar content with an average increase of 71.7%. In general exogenous GABA (1 and 2mM) had a positive effect on the accumulation of sugars in tomato leaves both in the absence and presence of chilling stress.

### Changes in Chilling-Induced Oxidative Damage and Root Thermo-Tolerance

**Figure 4:**
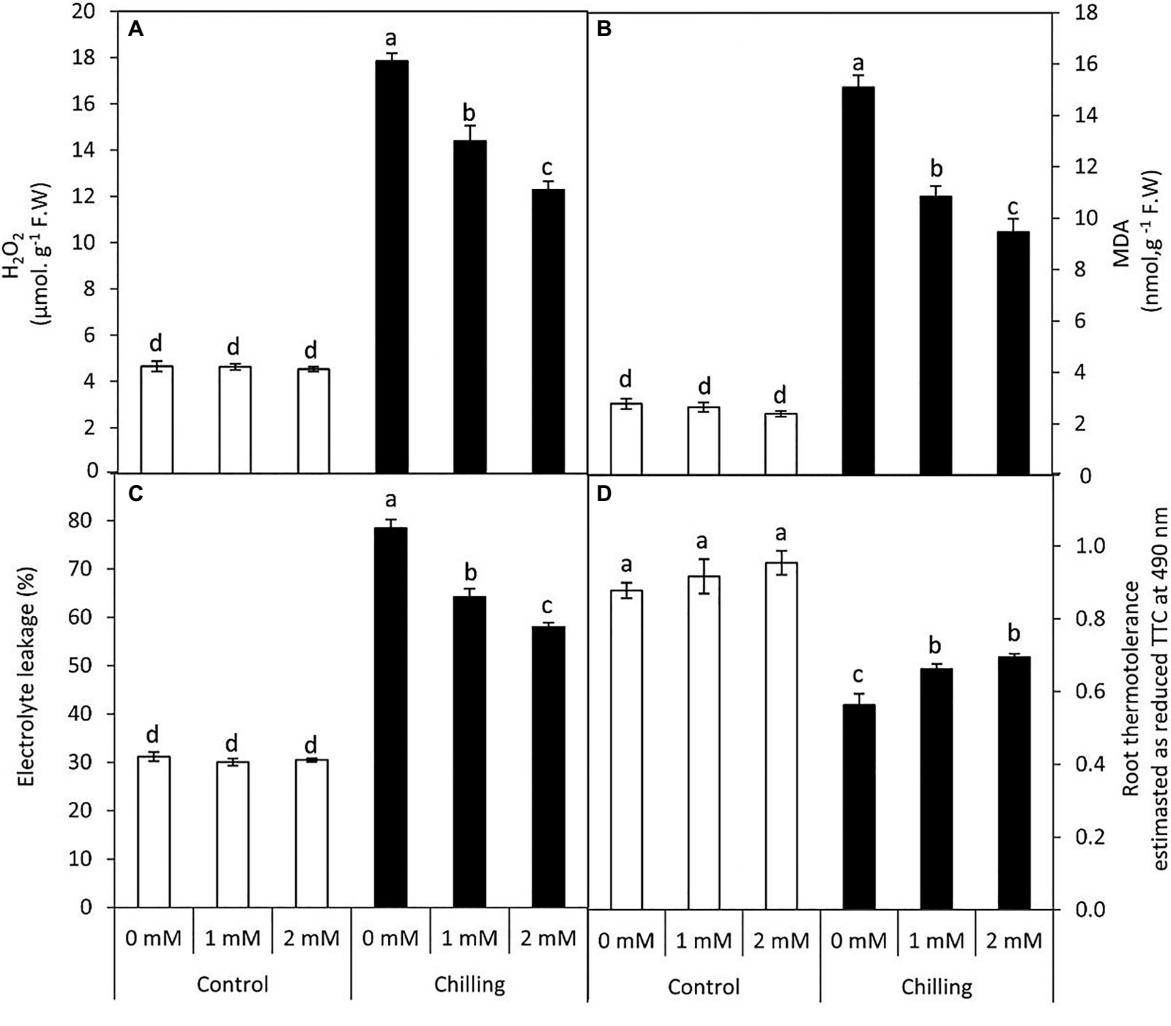
Effect of γ-aminobutyric acid (GABA) as a foliar application at 1 and 2mM on leaf oxidative damage and root tissues thermotolerance of tomato plants cv “Super marmende” growing under normal (25°C/20°C day/night ) and chilling conditions (5 days/4ºC for 6h/day) followed by 2 days recovery. **(A)**, hydrogen peroxide (H_2_O_2_); **(B)**, malondialdehyde (MDA); **(C)**, electrolyte leakage, and **(D)**, root thermotolerance as reduced TTC. Data followed by the same letters ± SE are not significant according to Duncan multiple rang test *P*≤0.05. The white columns refer to the control conditions and the black columns refer to the chilling stress.

Chilling stress in tomato plants resulted in a significant (*p*≤0.05) increase in reactive oxygen species (H_2_O_2_ content), membrane lipid peroxidation (MDA content), and electrolytic leakage, while a significant decrease in root thermo-tolerance was observed (Figures 4A–D). The accumulation of H_2_O_2_, MDA reached 5.4, 3.8–fold, respectively over the unstressed control plants. However, GABA application (1 and 2mM) during chilling stress resulted in a steady decrease in the extent of H_2_O_2_, lipid peroxidation (MDA) and electrolytic leakage, respectively. However, control plants subjected to GABA treatment did not exhibit any significant changes in these parameters. In contrast, root thermo-tolerance exhibited a marginal increase upon GABA application under chilling stress.

### Changes in the Antioxidant and Phenolic Related Enzymes

**Figure 5:**
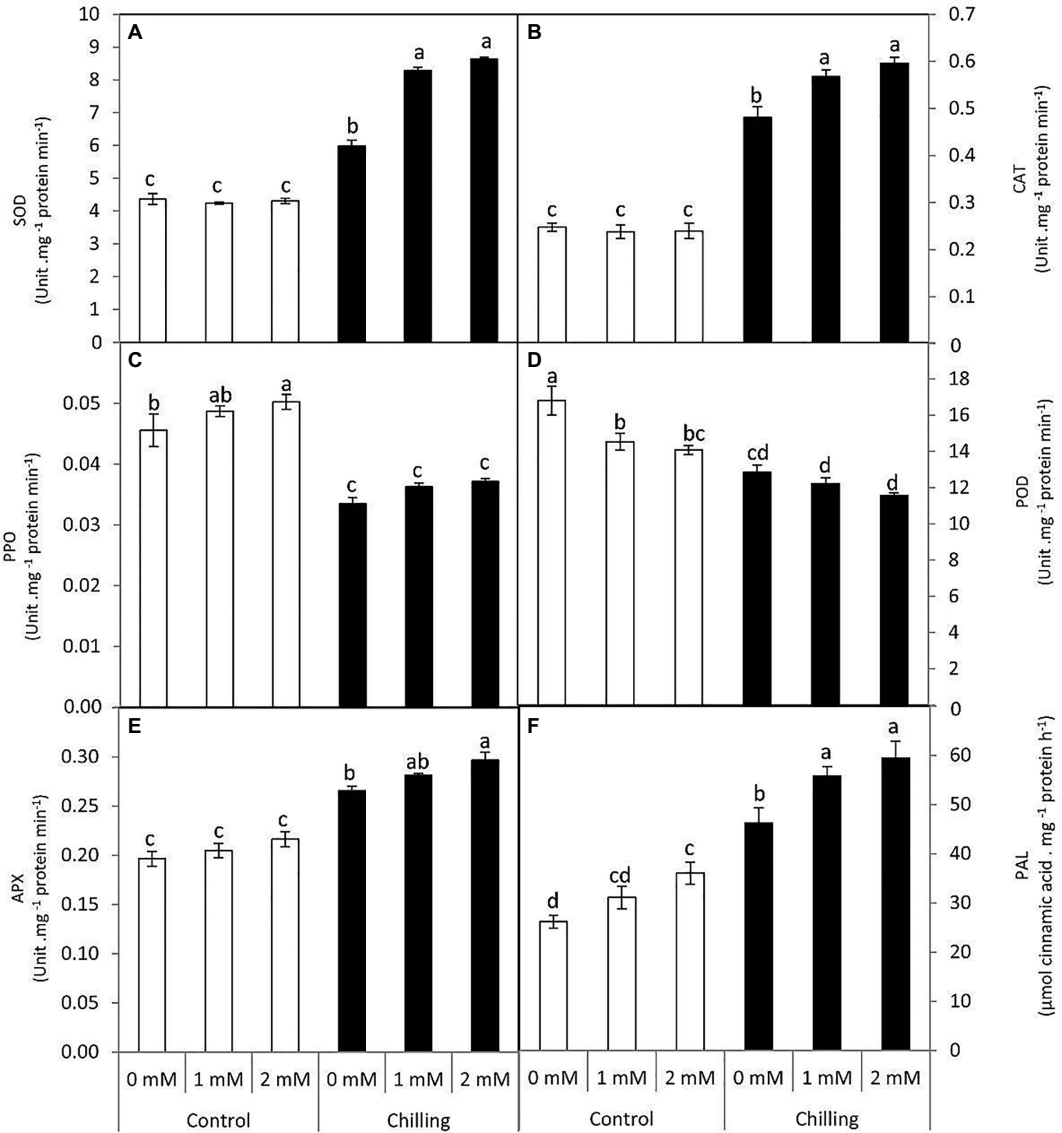
Effect of γ-aminobutyric acid (GABA) as a foliar application at 1 and 2mM on antioxidant and phenolic-related enzymes of tomato plants cv “Super marmende” growing under normal (25°C/20°C day/night ) and chilling conditions (5 days/4ºC for 6 h/day) followed by 2 days recovery. **(A)**, superoxide dismutase (SOD); **(B)**, catalase (CAT); **(C)**, polyphenol oxidase (PPO); **(D)**, peroxidase (POD); **(E)**, ascorbate peroxidase (APX), and **(F)**, phenylalanine ammonia lyase (PAL). Data followed by the same letters ± SE are not significant according to Duncan multiple rang test at *P*≤0.05. The white columns refer to the control conditions and the black columns refer to the chilling stress.

Superoxide dismutase and CAT activity were increased in the presence of chilling stress wherein catalase activity was elevated to almost 1.5-fold in comparison with control (Figures 5A,B). Under chilling stress, the exogenous GABA treatments, particularly 2mM, enhanced SOD and CAT activities, with increases of 97.7 and 140%, respectively. PPO activity was also analyzed during chilling stress and GABA application in tomato leaves (Figure 5C). Chilling stress significantly resulted in a decrease in PPO activity in comparison with control. PPO activity did not exhibit any significant changes in the presence of GABA application during chilling stress. Peroxidase activity (POD) was negatively upregulated in the presence of chilling stress and GABA application, respectively (Figure 5D). However, APX activity showed a gradual increase by the average of 35.2, 43.2, and 51%, respectively in the presence of chilling stress and GABA treatments (Figure 5E). PAL activity showed a noticeable increase in the presence of chilling stress (Figure 5F). Exogenous GABA positively upregulated PAL activity both in the absence and presence of chilling stress.

### Relationship Between the Form of Soluble Carbohydrates and Secondary Metabolism

**Figure 6:**
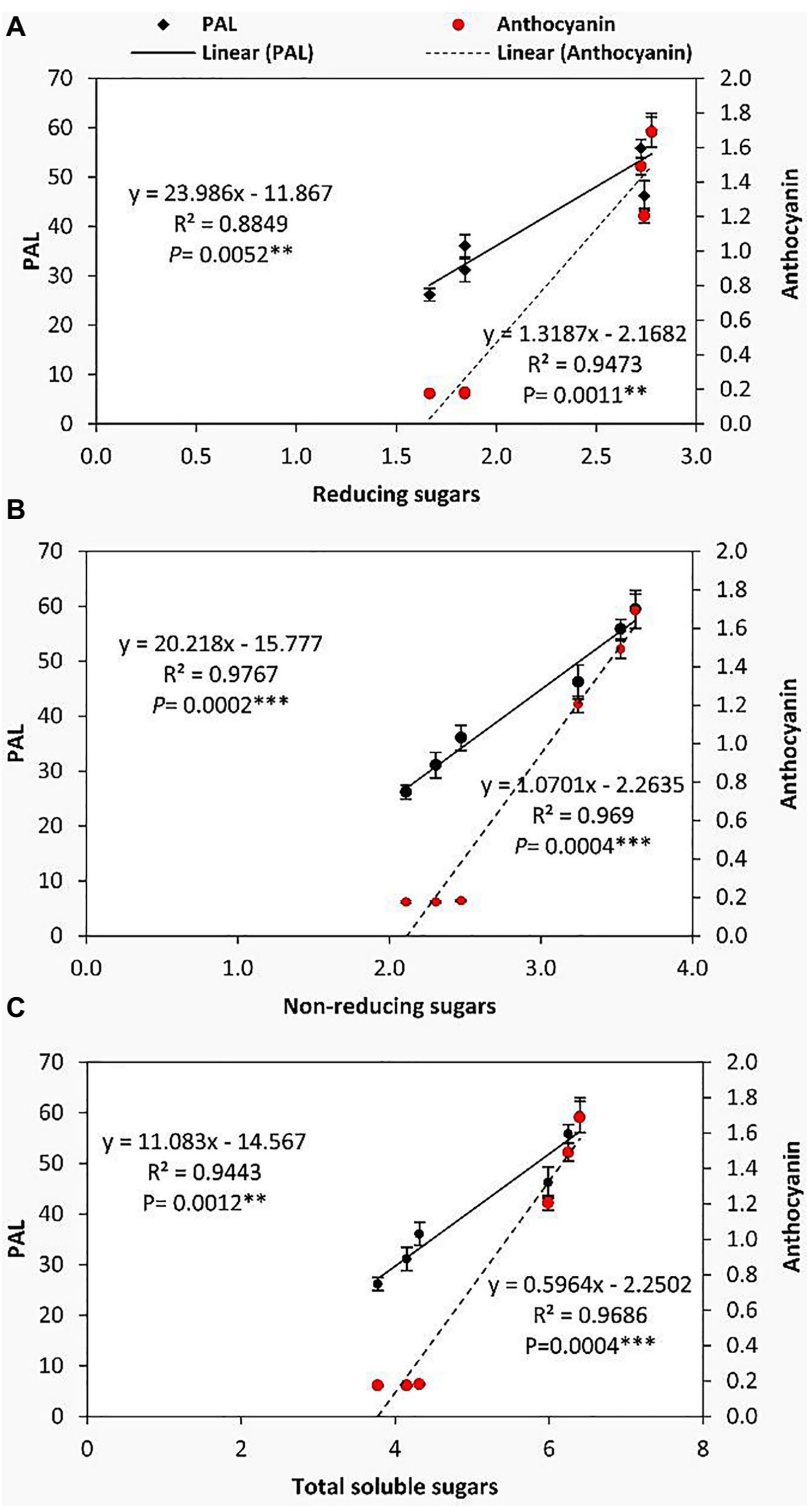
Linear regression analysis elucidates the relationship between the soluble sugars, PAL enzyme activity, and anthocyanin contents in tomato plants cv “Super marmende” treated by γ-aminobutyric acid (GABA) as a foliar application at 1 and 2mM and growing under normal (25°C/20°C day/night) and chilling conditions (5 days/4ºC for 6h/day) followed by 2 days recovery. ns. non-significant; ^*^P≤0.05; ^**^P≤0.01; and ^***^P≤0.001.

To further illuminate the relationships between the accumulation of soluble sugars and the secondary metabolism of GABA-treated and untreated tomato plants under chilling stress, linear regression analysis was performed (Figure 6). It can be observed that PAL and anthocyanin were positively and significantly correlated with the accumulation of reducing sugars, non-reducing sugars, and total soluble sugars in leaves. Precisely, PAL was more correlated with the non-reducing sugars (*R*^2^=0.9767, *p*=0.0002) than reducing and total soluble sugars, while anthocyanin exhibited a highly significant correlation with non-reducing sugars (*R*^2^=0.969, *p*=0.0004) and total soluble sugars (*R*^2^=0.9686, *p*=0.0004) compared to the reducing sugars. These results specifically imply that in the flowering stage, GABA-pretreatment may induce its protective effect against chilling stress in tomato plants through a close linkage between the transformation of soluble carbohydrates to non-reducing form (the major transport form) and the simultaneous upregulation of flavonoid pathway (secondary metabolism).

### Relationship Between the Form of Soluble Carbohydrates and Antioxidant Enzymes

**Figure 7:**
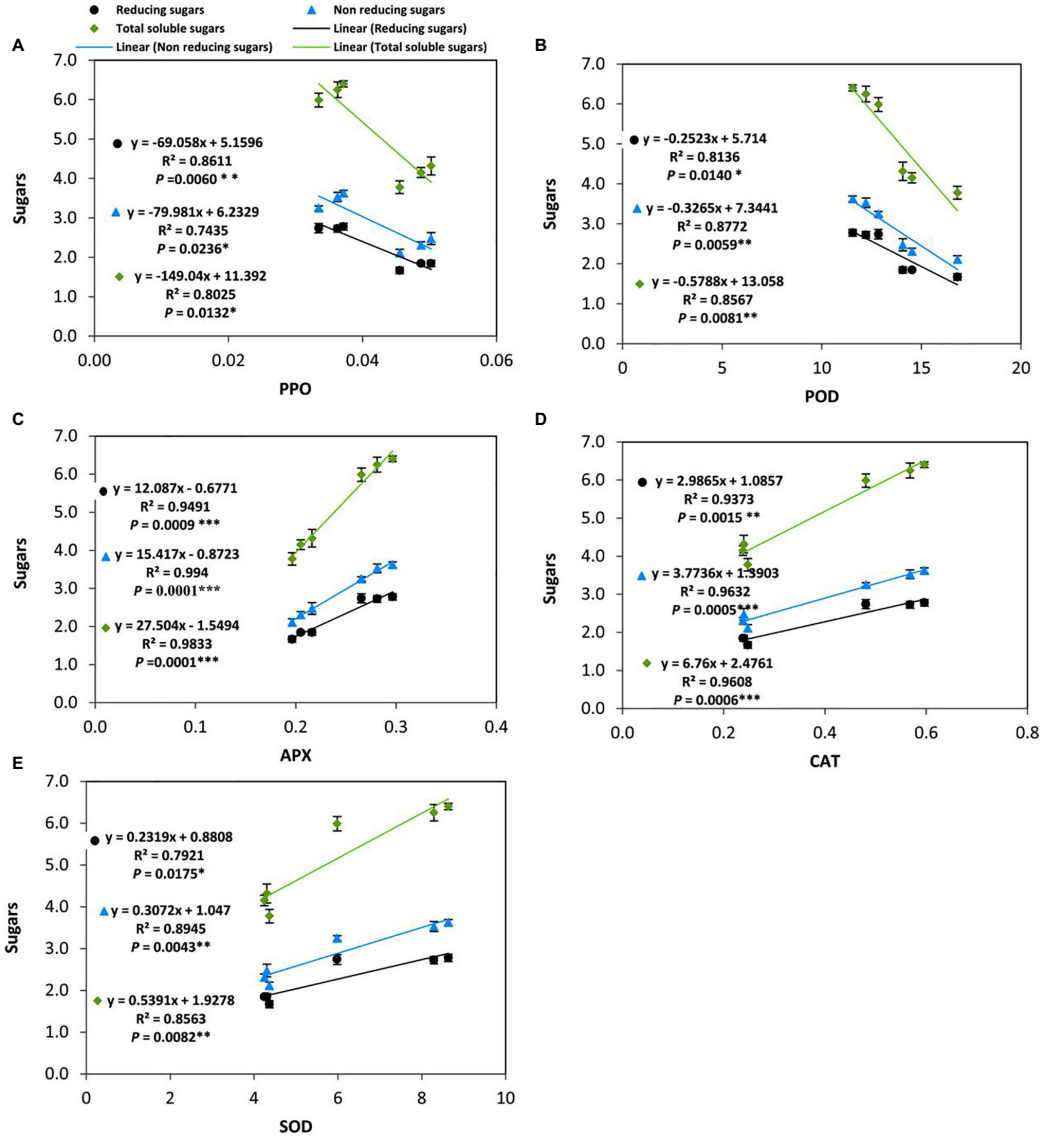
Linear regression analysis elucidates the relationship between the soluble sugars, antioxidant enzyme activities, superoxide dismutase (SOD), catalase (CAT), polyphenol oxidase (PPO), and peroxidase (POD), ascorbate peroxidase (APX) in tomato plants cv “Super marmende” treated by γ-aminobutyric acid (GABA) as a foliar application at 1 and 2mM and growing under normal (25°C/20°C day/night) and chilling conditions (5 days/4ºC for 6 h/day) followed by 2 days recovery. ns. non-significant; ^*^P≤0.05; ^**^P≤0.01; and ^***^P≤0.001.

Several previous studies have reported that soluble carbohydrates can play an important role in reducing cold-induced oxidative damage in plants. To gain further insights into the effect of applied-GABA on chilling-stressed tomato plants during the reproductive stage, we analyzed the relationships between the accumulation of different forms of soluble sugars (Reducing, non-reducing, and total soluble sugars) and the activities of antioxidant enzymes in leaves (Figure 7). The results indicated that PPO and POD negatively and significantly correlated with different forms of soluble sugars which mean PPO and POD did not contribute to control the level of ROS in the cold leaf stressed tissues of tomato plants, while positive and significant correlations were observed with APX, CAT, and SOD in this respect. More precisely, POD (*R*^2^=0.8772, *p*=0.0059), CAT (*R*^2^=0.9632, *p*=0.0005), and SOD (*R*^2^=0.8945, *p*=0.0043) were highly correlated with non-reducing sugars than the reducing ones; Whereas, APX was strongly correlated with both reducing and non-reducing sugars at the same level of significance (*p*≤0.001). On the other hand, PPO exhibited an obvious negative correlation with total sugars (*R*^2^=0.8611, *p*=0.0060) more than any individual type of sugars.

### Changes in the Fruit Yield

**Figure 8:**
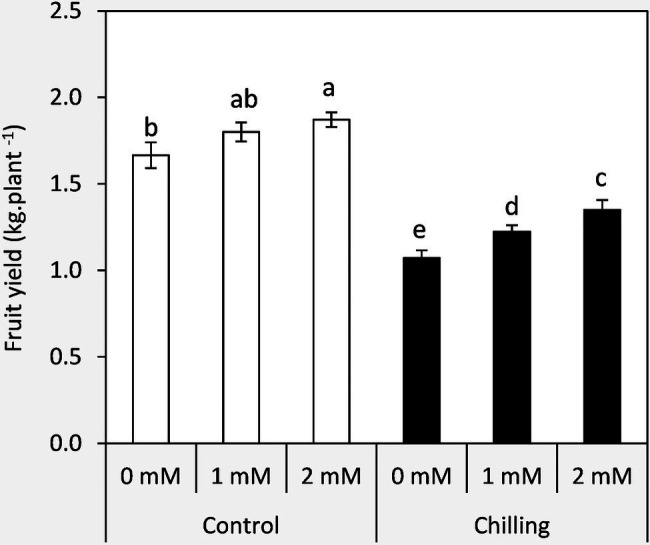
Effect of γ-aminobutyric acid (GABA) as a foliar application at 1 and 2mM on fruit yield of tomato plants cv “Super marmende” growing under normal (25°C/20°C day/night ) and chilling conditions (5 days/4ºC for 6h/day) followed by 2 days recovery. Data followed by the same letters ± SE are not significant according to Duncan multiple rang test at *P*≤0.05. The white columns refer to the control conditions and the black columns refer to the chilling stress.

Plants exposed to chilling stress demonstrated a significant decrease in fruit yield compared to the unstressed plants by an average of 64.3% (Figure 8). Plants treated by GABA especially at 2mM achieved the highest significant increases in the fruit yield in both chilling stress and non-stressed plants. These results indicate the possible role of GABA in fruit set and mitigation of low temperatures in tomato plants.

### Exogenous GABA Application Alleviates Chloroplast Damage and Maintains Starch Grain Integrity in Chilling-Stressed Tomato Leaves

Tomato leaves grown under standard conditions at 25°C have a normal green color. Whereas leaves exposed to chilling stress changed color from green to dark red or purple. The red color appeared firstly on the leaf margins and gradually spread throughout the leaf surface, the major and minor veins gaining the blue or purple colors.

**Figure 9:**
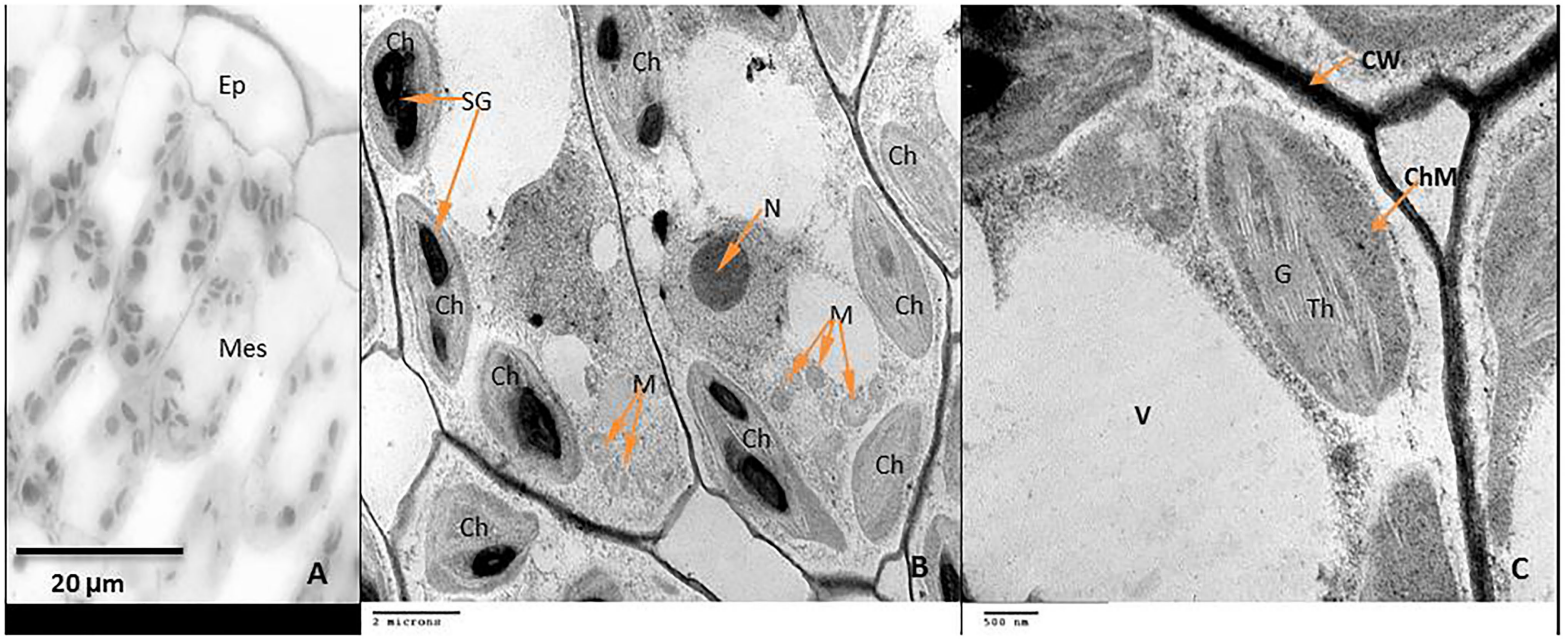
Anatomical structure of Tomato leaf cv “Super marmende” growing under standard condition 25ºC. **(A)**. Part of leaf cross-section showed the upper epidermis (Ep) and the two mesophyll layers (Mes); note the regular arrangement of chloroplasts. **(B)**. TEM micrograph showed mesophyll ultra-structures: Mitochondria (M), nucleus (N), chloroplasts (Ch) with elongate shape, and starch grains (SG). **(C)**. Ultra-structure of one chloroplast showed it has normal envelope or membrane (ChM), well-developed granal lamella (G), and stromal thylakoids (Th), note that the outer cellular membrane was attached well with the cell wall (CW).

**Figure 10:**
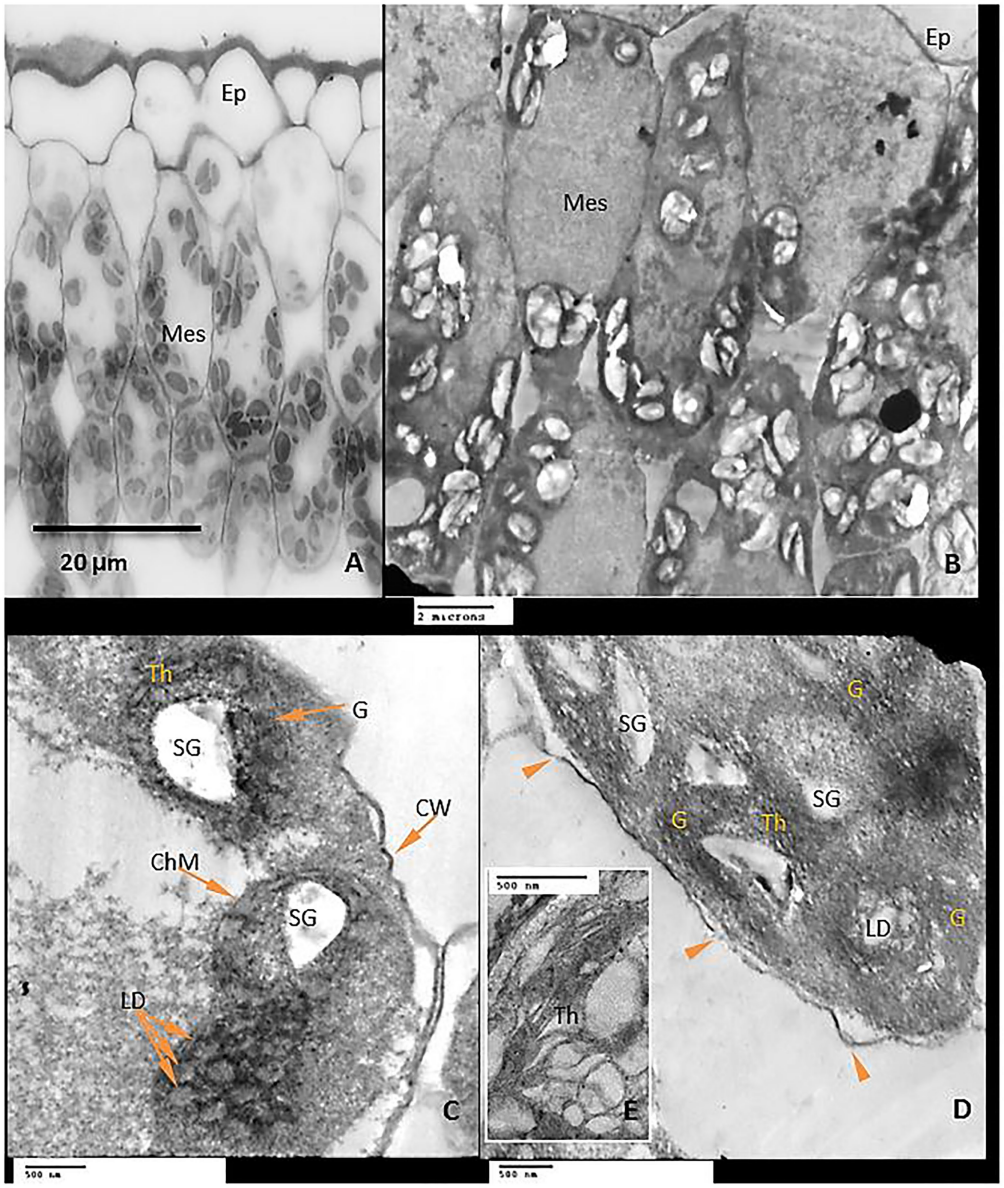
Anatomical structure of Tomato leaf cv “Super marmende” growing under chilling conditions 5 days/4ºC for 6h/day. **(A)** Leaf cross-section showed the upper epidermis (Ep) and the two mesophyll layers (Mes); note that chloroplasts were accumulated together in clusters. **(B)** TEM micrograph showed mesophyll ultra-structures: Chloroplasts became swollen with more rounded shape. **(C)** High magnification of two chloroplasts showed large starch spherical grains (SG) with accumulation of lipid droplets (LD) in stroma and the membranes of granal (G) and stromal thylakoids (Th) were indistinct. **(D,E)**, note the unstacking of grana and dilation of thylakoids. Arrowheads pointed to disintegration the outer cellular membrane which disconnected partly from the cell wall.

Examination of leaf anatomy under control conditions revealed that the leaf consists of the common epidermal layers enclosed in between the mesophyll, which is intervened by some vascular bundles (Figure 9A). Mesophyll ultra-structures pointed out that the outer cell membrane was attached well with the cell wall, organelles could be observed clearly; chloroplasts, nucleus, mitochondria as well as the vacuole (Figure 9B). The chloroplasts distribute regularly and have an elongated or ellipsoidal shape with distinct normal envelopes. It was characterized by well-developed grana and stromal thylakoids. Sometimes, starch grains appeared with acceptable size (Figures 9B,C). During chilling, stress plastids are affected more than other organelles. It aggregated together in clusters and became swollen with a more rounded shape compared to ellipsoidal recorded in control plants (Figures 10A,B). The chloroplast envelope was disintegrated or completely disappeared. The starch grains became larger, with a spherical shape; the membranes of granal and stromal thylakoids were indistinct, with a noticeable accumulation of lipid droplets in the dark stroma (Figure 10C). Sometimes, the outer cellular membrane ruptured and partially disconnected from the cell wall (Figures 10D,E).

**Figure 11:**
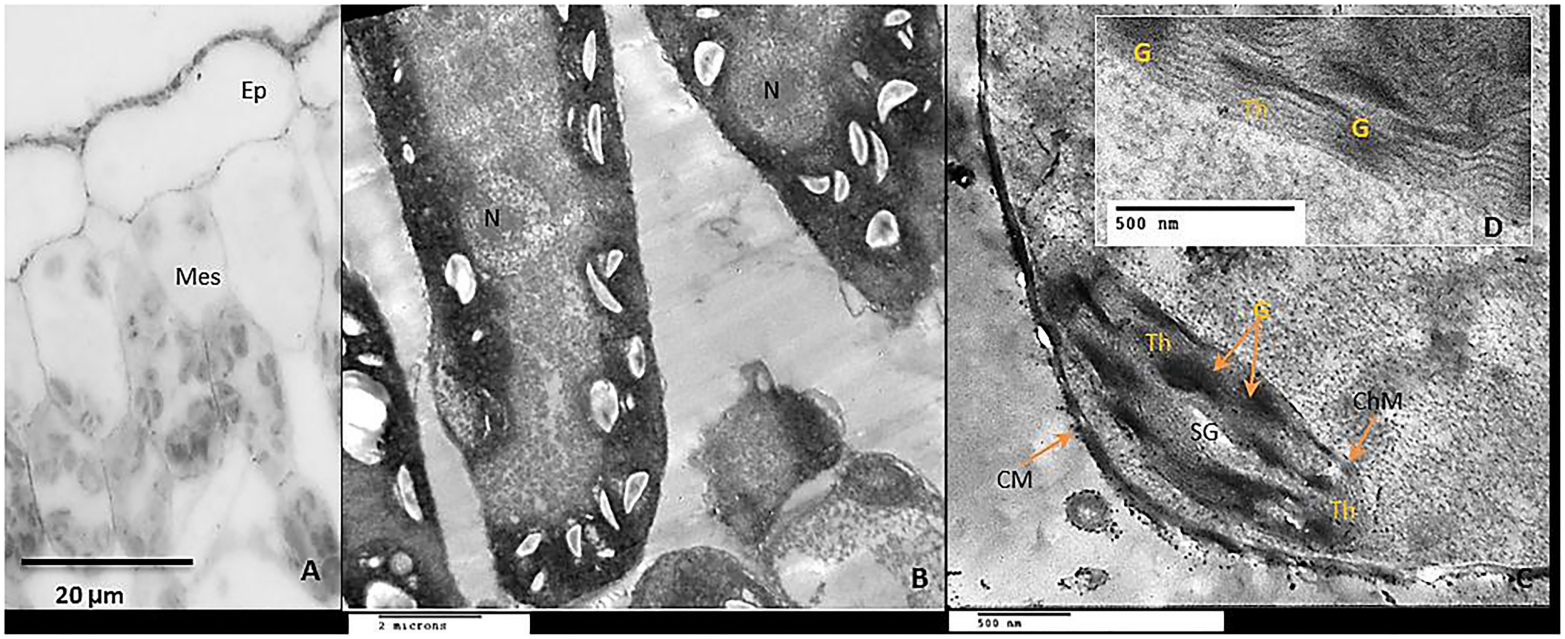
Anatomical structure of Tomato leaf cv “Super marmende”growing under stress chilling conditions 5 days/4ºC for 6h/day treated with GABA at 2mM. **(A)** Leaf cross-section showed the upper epidermis (Ep) and the two mesophyll layers (Mes); note that chloroplasts were accumulated together in clusters. **(B)** TEM micrograph showed mesophyll ultra-structures: There are expansions in the size of the plastids due to the large starch grains in them, but with a tendency to elongate shape. **(C,D)** high magnification of one chloroplast showed normal chloroplast membrane (ChM), well-developed grana (G), and stromal thylakoids (Th), the cell wall (CW) was attached well with the outer cellular membrane.

Exogenous GABA application reduced chloroplast damage caused by chilling stress by recovery of the plastid shape, starch grains were observed in smaller size and as having an elongated shape, granal lamellae were better integrated than those from chilling stress in untreated plants (Figures 11A–D).

## Discussion

The present work provides evidence on the beneficial role of GABA treatment (foliar spray) on tomato plants subjected to chilling stress. The tomato plants were subjected to GABA pre-treatment (50–56days) at the initial stage of the reproductive phase, which was characterized by the emergence of floral bud. The reproductive stage of tomato plants is associated with rapid translocation of organic solute to the emerging floral buds and flowers in the pre and post-pollination phase. Followed by GABA application the tomato plants were subjected to chilling stress for 5days at an age of 60–64days. Interestingly, GABA application resulted in modulation of pigment composition, secondary metabolism, sugar accumulation, and antioxidative defense during chilling stress exposure at a later stage. Thus, GABA application appears to be a good priming option for developing tolerance to chilling stress in tomato plants prior to the fruiting stage.

Current findings reveal that exogenous GABA (2mM) exerts differential effects on chl-a and chl-b content in the presence of chilling stress. Exposure of tomato plants to chilling stress resulted in a decrease in both chl a and chl b content in leaves, while plants pretreated with exogenous GABA exhibited a significant increase in chl b content. Exogenous GABA is an effective priming molecule that is known to protect the structure and function of the PSII center in the chloroplast of musk melon plants subjected to alkalinity and salinity stress (Xiang et al., 2016). Although chl b content did not exhibit significant changes after chilling stress, GABA-induced increase in chl b content is attributed to modulation of photosynthetic capacity achieved by optimum light-harvesting efficiency. PSII functioning and net photosynthesis rate are known to be upregulated in response to GABA application during salinity stress in wheat plants (Li et al., 2016). Thus, GABA-treated tomato plants subjected to chilling stress (present work) are expected to exhibit improved photosynthetic efficiency. Chilling stress significantly increased anthocyanin content in the leaves of tomato plants which was all the more high in GABA-pretreated plants. However, GABA treatment did not exhibit any significant changes to chilling stress-induced reduction in carotenoid content. Anthocyanin accumulation in response to chilling stress and GABA-pretreatment indicate upregulation of flavonoid pathway (secondary metabolism) associated with tolerance to chilling stress. Although carotenoid content exhibits decreases due to chilling stress, a surge in chl b and anthocyanin is attributed to an increase in the light-harvesting efficiency of chilling stressed tomato leaves. A surge in anthocyanin biosynthesis and associated secondary metabolites has been known to be a tolerance mechanism to cold stress (Ahmed et al., 2015; Li et al., 2016; Sicilia et al., 2020). Apart from its role as a free radical scavenger, anthocyanin accumulation in the leaves of chilling stressed tomato plants (present work) is expected to be associated with reduced osmotic potential and delayed freezing of cells *via* surface nucleators (Chalker-Scott, 1999; Nagata et al., 2003).

Interestingly, GABA application appears to be a positive regulator of sugar accumulation (reducing and non-reducing) in the leaves of tomato plants both in the absence and presence of chilling stress. However, non-reducing sugars exhibited a significant increase in GABA-pretreated tomato leaves subjected to chilling stress. GABA-induced increments in non-reducing sugar coincided with changes in the shape of starch grains in the chloroplast of the leaves, as visualized by transmission electron microscopy. Chilling stress (in the absence of GABA) treatment exhibited clustered accumulation of chloroplast which was swollen and round in shape with indistinct thylakoid and filled with large-sized spherical starch grains. It is noteworthy that GABA pretreatment in chilling-stressed tomato leaves normalized the structure of the chloroplast and starch grains mostly appeared to be ellipsoidal in shape. An increase in both non-reducing and reducing sugars in the leaves after exposure to chilling stress depict upregulation of starch biosynthesis and sugar accumulation as osmolytes in cells, respectively. Optimum levels of carbon assimilation are preferably maintained by improved photosynthetic efficiency and increased transport of assimilates. Leaves are important source tissue that exhibit precise regulation of starch-sugar inter-conversion during abiotic stress signals (Thalmann and Santelia, 2017; Thitisaksakul et al., 2017). In the present work, GABA treatment induces a surge in starch accumulation which is likely to function as a reserve for inducing sucrose (osmolyte accumulation) formation in the later stages of flower and fruit development. Chilling stress brings about a significant increase in the reducing and soluble sugar content which, therefore, does not exhibit any significant increase in the presence of GABA treatment. GABA-induced increase in sugar content during abiotic stress depicts its role as an inducer of osmoprotectant (Wang et al., 2017). Osmotic stress results in alteration in the transport of organic assimilate (Yang et al., 2002; Rizhsky et al., 2004; Cramer et al., 2007) between the source and sink organs. In the present work, the leaves were obtained from plants with emerging floral buds. Thus, high levels of TSS after chilling stress indicate prior adaptive mechanisms for optimum transport and nutrient allocation in buds and floral parts. GABA-pretreatment resulted in a marginal increase in TSS. In addition to the function of starch as a storage molecule, it is known to exhibit transient changes during abiotic stress thus indicating its role in the metabolic fitness of plants (Pressel et al., 2006; Cuellar-Ortiz et al., 2008; Yin et al., 2010; Thalmann and Santelia, 2017). Starch degradation during abiotic stress is associated with improved osmotic tolerance attained by higher sugar accumulation. Thus, in the present work higher starch accumulation in GABA-treated chilling-stressed plants indicates signaling events preceding further osmolyte accumulation in leaves (Rezaei-Chiyaneh et al., 2018). Recent investigations from the author’s laboratory have also revealed GABA-induced accumulation of soluble sugars in drought-stressed snap bean leaves (Abd El-Gawad et al., 2020). GABA-induced abatement of nitrogen stress in green microalga *Tetraselmis sub cordiformis* has been known to be associated with increased starch accumulation (Ran et al., 2020).

γ-aminobutyric acid-induced alleviation of chilling stress in tomato leaves is evident from reduced lipid peroxidation (MDA content), which is in congruence with decreased electrolytic leakage. The present findings are in line with various earlier investigations which reported GABA-induced osmotic tolerance and reduced lipid peroxidation (Nayyar et al., 2014; Wang et al., 2017; Cheng et al., 2018; Abd El-Gawad et al., 2020). Furthermore, GABA-pretreated tomato plants subjected to chilling stress exhibited a marginal increase in root thermotolerance. Foliar application of GABA, therefore, seems to exert a long-distance signaling effect from foliage to roots by imparting thermotolerance during chilling stress. Chilling stress in tomato plants resulted in a significant increase in hydrogen peroxide content accompanied by increased catalase activity. Although hydrogen peroxide priming has been known to be beneficial for abiotic stress tolerance in plants (Hossain et al., 2015; Arfan et al., 2019) higher levels appear toxic to plant cells. Higher catalase activity in leaves of tomato plants subjected to chilling stress and GABA-pretreatment is also accompanied by upregulation of SOD and APX activity. Redox balance in leaves is crucial to maintain the optimum efficiency of metabolic enzymes and the electron-transport cascade of the photosystem in the chloroplast. Thus, increased chlorophyll b and anthocyanin content (responsible for light harvest efficiency) is associated with elevated antioxidative defense in the leaves of tomato plants subjected to GABA-pretreatment before chilling stress. Present findings are in congruence with earlier reports of GABA-induced elevation of SOD, CAT, and APX activity in chilling stressed-tomato seedlings (Malekzadeh et al., 2014). GABA has been known to function as an important regulator of ROS scavengers in plant systems (Nayyar et al., 2014; Vijayakumari and Puthur, 2016; Carillo, 2018). According to Cheng et al. (2018) GABA-induced antioxidative defense in white clover is mediated by upregulation of Cu/ZnSOD, MnSOD, FeSOD, GPOX, CAT, APX, MDHAR, GST, and GPX genes. In the present work, GABA-induced elevation in CAT and APX activity is accompanied by reduced POD activity during chilling stress. POD is represented by several isoforms in plant organs with a different k_m_ value for its substrate (hydrogen peroxide). Our findings reveal a negative correlation between CAT and POD activity during GABA treatment and wherein, hydrogen peroxide detoxification mostly appears to be catalyzed by CAT and APX activity.

Analysis of enzymes associated with phenol metabolism revealed differential regulation of chilling stress on the modulation of PPO and PAL activity. It is noteworthy that although chilling stress decreased the activity of PPO, a significant surge in PAL activity was observed in the leaves. PAL activity was all the more elevated in presence of GABA treatment. PPO is a crucial enzyme localized in the thylakoid lumen and known to be associated with the restoration of photosynthetic functions. Although PPO catalyzes the activity of vacuolar localized monophenols, further investigations are required to decipher its direct role in oxidative stress or the regulation of photosynthetic efficiency. However, higher PPO activity in chloroplasts might be associated with the protection of the electron transport mechanism (Boeckx et al., 2015). In the present work, GABA-induced elevation in antioxidative defense is associated with a reduction in PPO activity. A similar report of GABA-induced downregulation of PPO activity has been reported in chilling stressed mango fruits (Rastegar et al., 2020). Our findings are incongruent with reports of Rastegar et al. (2020), where reduced PPO activity is associated with elevated CAT activity. GABA application and chilling stress positively upregulated PAL activity in tomato leaves thus suggesting the possible involvement of pheynyl-propanoid pathways in chilling tolerance. Thus, in support of earlier evidence of GABA-mediated upregulation of PAL activity in banana and barley (Wang et al., 2014; Ma et al., 2018), present findings indicate events of chilling tolerance to be associated with PAL activity in tomato leaves.

In the present study, linear regression analysis under cold stress and GABA as a foliar application demonstrated a number of close linkages between the soluble carbohydrates (specifically the non-reducing sugars) and secondary metabolisms in respect of the upregulation of PAL activity and biosynthesis of anthocyanin. The primary metabolites of photosynthesis can play a crucial role in the secondary metabolism specifically the biosynthesis of flavonoid-based compounds. This effect can occur through two distinct pathways, including the shikimic acid pathway generating the phenylpropanoids (C6-C3) skeleton, and the acetate pathway that serving as a building block for polymeric 2-carbon units (Croteau et al., 2000). Furthermore, soluble sugars can function as signaling molecules or primary messengers in signal transduction (Yuanyuan et al., 2009), which gives them the ability to regulate cold-induced gene expression (Tabaei-Aghdaei et al., 2003).

Moreover, multiple correlations were detected between the soluble sugars (reducing/non-reducing) and the activity of antioxidant enzymes. Soluble sugars can protect plant cells from chilling stress through interacting with the lipid bilayer of membranes and serving as osmoprotectants (Yuanyuan et al., 2009). Additionally, soluble sugars have been reported to be involved in the balance of ROS and consequently the responses to oxidative damage (Couée et al., 2006). Several metabolic reactions related to the production of ROS in plant cells have been found to be directly correlated with soluble sugars i.e., photosynthesis and mitochondrial respiration. However, soluble sugars have been found to be involved in the anti-oxidative processes, such as the oxidative pentose-phosphate pathway and carotenoid biosynthesis (Couée et al., 2006). In this study, the author found that POD, CAT, and SOD were highly correlated with non-reducing sugars than the reducing ones. These results may be related to activate sucrose-specific signaling pathways by GABA application.

The structure of mesophyll cells, including the chloroplasts, is the main component for the photosynthesis process and play a crucial role in determining the photosynthetic assimilation capacity (Araki et al., 2012). Under stress conditions, the morphology and ultrastructure of chloroplasts are directly disturbed causing a significant decrease in photosynthetic efficiency, accumulation of dry matter, and loss of crop yield (Weston et al., 2000; Shao et al., 2014). Previous studies confirmed that the chloroplasts in plants subjected to abiotic stress are considered the primary sites for generating ROS (Sun et al., 2002; Gill and Tuteja, 2010). Our results showed that chilling stress affects chloroplast ultrastructure including swollen and abnormal shape, disintegration of the chloroplast envelope or complete disappearance, and dilation of thylakoids. Over time, small lipid droplets accumulated in chloroplast (Figures 10A–E). These results were coincided with a reduction in chlorophyll content (Figures 2A–D). At the same time, chloroplast ultrastructure might be damaged by chilling stress resulting from accumulation of ROS (Figures 4A,B,D). Changes in thylakoid membranes with the slowing in enzymatic reactions caused by chilling stress directly affect chlorophyll content and photosynthetic activities (Liu et al., 2018). However, GABA application measurably reduced chilling injury by protecting thylakoid membranes, chloroplast envelopes, and diminishing swelling (Figures 11A–D). This, in turn, improves the photosynthesis process *via* alleviating chlorophyll degradation (Figures 2A–D). In addition, chilling injury affects membranes by disintegrating the outer cellular membrane which is disconnected partly from the cell wall (Figure 10D). This was synchronized with increasing in H_2_O_2_, MDA, and electrolyte leakage contents, which are regarded as biochemical markers for the occurrence of ROS under chilling stress (Figure 4A). Whereas, exogenous application of GABA kept the stability of membranes *via* reducing the construction of lipid peroxidation products (Figure 4B). In addition significant enhancement of the activities of SOD, CAT, APX, and PAL antioxidant enzymes which create a defense system against chilling stress and protect membrane damage (Figures 5A–F) was noted.

To sum up, the present work provides a correlation between GABA-induced alleviation of chloroplast damage and the modulation of pigment composition in chilling stressed tomato leaves (Figure 7). Furthermore, increased chl b and anthocyanin content is accompanied by elevated starch levels and improved osmotic tolerance evident from reduced MDA content and electrolytic leakage. Further investigations are required to decipher the detailed role of GABA in the modulation of carbohydrate and phenol metabolism in tomato plants subjected to chilling stress.

## Data Availability Statement

The raw data supporting the conclusions of this article will be made available by the authors, without undue reservation.

## Author Contributions

OE, AE, and MI: conceptualization. OE, AE, GN, TW, RF, SM, AA-H, HE-H, AE-Y, HE-G, EA, AG, NN, AE-S, AB, and MI: methodology, validation, resources, and writing – review and editing. OE, SM, GN, TW, AA-H, EA, AG, NN, AE-S, AB, and MI: software. OE, AE, RF, SM, AA-H, HE-H, NN, AE-S, AB and MI: formal analysis. RF, SM, AA-H, HE-H, AE-Y, HE-G, EA, AG, NN, AE-S, and AB: investigation. OE, AE, GN, TW, RF, SM, and MI: data curation. OE, SM, and MI: writing – original draft preparation. RF, SM, AA-H, HE-H, AE-Y, HE-G, EA, AG, NN, AE-S, AB, and MI: supervision. AE-Y, GN, TW, HE-G, MI, SM, AE-Y, EA, HE-G, AG, RF, and NN: project administration. OE, AE, GN, TW, EA, AG, RF, and MI: funding acquisition. All authors contributed to the article and approved the submitted version.

## Acknowledgments

We thank Taif University Researchers Supporting Project number (TURSP -2020/13), Taif University, Taif, Saudi Arabia.

## Conflict of Interest

The authors declare that the research was conducted in the absence of any commercial or financial relationships that could be construed as a potential conflict of interest.

## Publisher’s Note

All claims expressed in this article are solely those of the authors and do not necessarily represent those of their affiliated organizations, or those of the publisher, the editors and the reviewers. Any product that may be evaluated in this article, or claim that may be made by its manufacturer, is not guaranteed or endorsed by the publisher.
