# Erratum: Protective Effect of γ-Aminobutyric Acid Against Chilling Stress During Reproductive Stage in Tomato Plants Through Modulation of Sugar Metabolism, Chloroplast Integrity, and Antioxidative Defense Systems

**DOI:** 10.3389/fpls.2021.812646

**Published:** 2021-11-19

**Authors:** Frontiers Production Office

**Affiliations:** Frontiers Media SA, Lausanne, Switzerland

**Keywords:** tomato (*Solanum lycopersicum* L.), gamma-aminobutyric acid, chilling stress, chloroplast ultrastructure, oxidative stress, antioxidants, fruit yield

Owing to a production error, the author Nihal El Nahhas was not included in the original article when published.

In addition, the contributions of the authors Gniewko Niedbała and Tomasz Wojciechowski were not included in the *Author Contributions* section of the original article when published. The correct *Author Contributions* section is presented here:

“OE, AE, and MI: conceptualization. OE, AE, GN, TW, RF, SM, AA-H, HE-H, AE-Y, HE-G, EA, AG, NN, AE-S, AB, and MI: methodology, validation, resources, and writing – review and editing. OE, SM, GN, TW, AA-H, EA, AG, NN, AE-S, AB, and MI: software. OE, AE, RF, SM, AA-H, HE-H, NN, AE-S, AB, and MI: formal analysis. RF, SM, AA-H, HE-H, AE-Y, HE-G, EA, AG, NN, AE-S, and AB: investigation. OE, AE, GN, TW, RF, SM, and MI: data curation. OE, SM, and MI: writing – original draft preparation. RF, SM, AA-H, HE-H, AE-Y, HE-G, EA, AG, NN, AE-S, AB, and MI: supervision. AE-Y, GN, TW, HE-G, MI, SM, AE-Y, EA, HE-G, AG, RF, and NN: project administration. OE, AE, GN, TW, EA, AG, RF, and MI: funding acquisition. All authors contributed to the article and approved the submitted version.”

The publisher apologizes for these mistakes. The original article has been updated.

